# A New Preparation of Gold for Filling Teeth

**Published:** 1850-10

**Authors:** 


A *New Preparation of Gold for Filling Teeth.*—Within the last few weeks several dentists in New York, have tested the value of sponge gold, as a substitute for gold foil in filling teeth, but to what conclusion they have arrived with regard to it, we have not been able to ascertain. We have been informed, however, that some are disposed to think very favorably of it.

The preparation is of a reddish brown color, and readily crumbles into a fine powder on being rubbed, but on being pressed together, it at once becomes a compact mass. A few weeks ago, we received from the manufacturer, Dr. Maire, through Dr. G. E. Hawes, of New York, a small quantity of it, with a request that we would try it. This we have done, but our experiments as yet are too limited to enable us to say much with regard to its value. We tried it in the cavity of a dead tooth out of the mouth, and succeeded in making a very solid filling. The surface assumed the color of gold and presented a highly polished and beautiful appearance, but on removing it from the tooth, we found that every other part retained the reddish brown color of the sponge, with here and there the lustre of a particle of gold. For front teeth, this would constitute an insuperable objection to its use. On breaking it, the fractured surfaces had the same appearance. We have also seen two incisors that were filled with it which were considerably discolored.

It may, perhaps, answer a very good purpose for filling a cavity in the grinding surface of a molar tooth, but as it crumbles into powder so readily we should not think it could be employed for filling a small cavity in the approximal surface of any tooth where the aperture between it and the adjoining organ was very narrow. It is possible, however, that some may have discovered a method of introducing it into the cavity of a tooth of which we are ignorant, and as our experiments with it have been very limited, we would be glad if some of our brethren of New York, who have given it a thorough trial, would favor us with their opinion with regard to its value.

